# Treatment of glenohumeral instability in rugby players

**DOI:** 10.1007/s00167-015-3979-8

**Published:** 2016-01-19

**Authors:** Lennard Funk

**Affiliations:** Upper Limb Unit, Wrightington Hospital, Hall Lane, Appley Bridge, WN6 9EP UK

**Keywords:** Rugby, Shoulder, Instability, Sport, Collision

## Abstract

Rugby is a high-impact collision sport, with impact forces. Shoulder injuries are common and result in the longest time off sport for any joint injury in rugby. The most common injuries are to the glenohumeral joint with varying degrees of instability. The degree of instability can guide management. The three main types of instability presentations are: (1) frank dislocation, (2) subluxations and (3) subclinical instability with pain and clicking. Understanding the exact mechanism of injury can guide diagnosis with classical patterns of structural injuries. The standard clinical examination in a large, muscular athlete may be normal, so specific tests and techniques are needed to unearth signs of pathology. Taking these factors into consideration, along with the imaging, allows a treatment strategy. However, patient and sport factors need to be also considered, particularly the time of the season and stage of sporting career. Surgery to repair the structural damage should include all lesions found. In chronic, recurrent dislocations with major structural lesions, reconstruction procedures such as the Latarjet procedure yields better outcomes. Rehabilitation should be safe, goal-driven and athlete-specific. Return to sport is dependent on a number of factors, driven by the healing process, sport requirements and extrinsic pressures.

*Level of evidence* V.

## Introduction

Rugby is the ninth most popular sport in the world, developed in England and now widely played in France where it has a strong tradition in the Basque, Occitan and Catalan people along the border regions between Spain and France. The game is very popular in South Africa, Australia and New Zealand. It has spread thence to much of Polynesia, having particularly strong followings in Fiji, Samoa and Tonga. Rugby is gaining popularity in Europe, South and North America. It is a high collision sport, with the highest incidence of traumatic injuries of all sports [13, 35]. Shoulder injuries are second to knee injuries, but result in the longest period out of play compared to any other injury [11]. As rugby players get larger and faster, the impacts involved have increased, despite changes in the rules to try to limit serious injuries [17]. It has been shown that the forces in modern-day rugby are in excess of 10 Gs, equivalent to low-impact motor vehicle accidents. This has led to more serious injuries than before, sometimes life-threatening. In the shoulder, varying degrees of instability are very common. These range from painful subclinical micro-instability to complete fracture-dislocations of the glenohumeral joint [16]. The direction of instability may be anterior, posterior, inferior or combined with a complex array of pathologies [10].

## Aetiology

The most common mechanism of shoulder injury is due to the tackling event [5]. Injuries can occur to the tackler or the ball carrier [18]. Video analysis studies have shown that the common mechanisms of injury result in predictable patterns of shoulder pathology. This information is useful in understanding the expected injuries prior to investigations. The common mechanism of injury are the tackle, ‘try-scorer’, direct impact and flexed fall [10].

1. Tackler injury

This occurs most commonly when the player tackles an opponent travelling towards them. The arm is held abducted to ninety degrees. A posteriorly directed force results from contact with the ball carrier. The tacklers’ arm extends behind the player in the plane of abduction, exerting a levering force on the glenohumeral joint.

Anterior dislocation is most common in tacklers, with a high incidence of anterior–inferior labral tears, SLAP tears and Hill–Sachs lesions. Humeral avulsions of the anterior band of the inferior glenohumeral ligament (HAGL) tend to be more common in tackling injuries. We have also found an increasing incidence of bony Bankart lesions recently [10].

2. Try-scorer injury

This mechanism occurs whilst diving and reaching the ball-carrying hand forward to score a try. The mechanism involves the injured arm in extreme overhead flexion above 90°. A posterior force drives the arm backwards and exerts leverage on the glenohumeral joint with the arm either remaining in fixed flexion by contact with the ground or forced into further hyper-flexion. This may be compounded by opposing players falling on top of the injured player, increasing the leverage on the glenohumeral joint.

The glenohumeral joint may subluxate or dislocate, resulting in Bankart tears, Hill–Sachs lesions and rotator cuff tears. This mechanism has a higher incidence of significant rotator cuff tears than the others [10].

3. Direct impact injury

The ball carrier may impact directly with the ground or another player, sustaining a large impact to the lateral aspect of the shoulder. The arm is held flexed below ninety degrees or neutral, with internal rotation, such as when carrying a ball by the side. A medially directed compressive force caused by direct impact to the shoulder results in injury.

Due to the variability of the exact impact vector, multiple complex injuries are sustained. This includes a combination of bony glenoid lesions, complex labral tears, scapula fractures and acromio-clavicular joint (ACJ) injuries.

4. Flexed fall injury

**Fig. 1:**
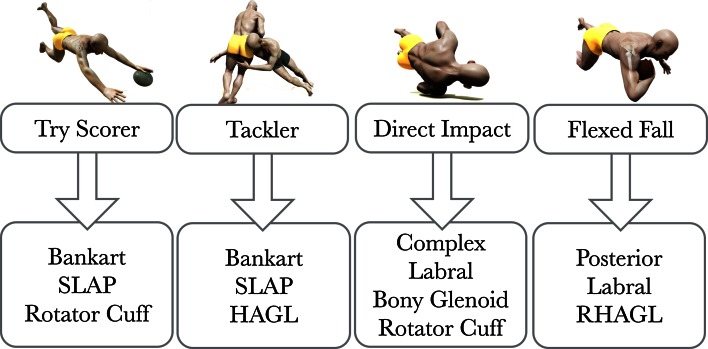
Common mechanism of shoulder injury in rugby and the structural injuries [10]

In rugby league, increasingly in modern rugby union, the ball carrier is tackled and lifted from behind. The ball-carrying elbow makes the first impact with the ground, with the elbow and shoulder joints flexed whilst holding the ball. This results in a large posterior directed force through the glenohumeral joint, causing injury and disruption to the posterior shoulder structures. The common pathologies are posterior labral tears, reverse bony Bankart lesions, reverse HAGL tears and posterior rotator cuff injuries (Fig. 1).

## Diagnosis

Instability in rugby players presents with a myriad of symptoms. It is easy to make the diagnosis where there has been frank dislocations and ongoing feelings of instability or recurrent dislocations. However, this is not often the case. Since 60–70 % of shoulder stability is muscular, most rugby players only get symptoms with extreme forces and activities due to their excellent abilities to compensate with their strong shoulder musculature. The usual clinical tests for instability may all be normal. A focused and thorough history and examination, tailored to the athlete, are required. There are a few common presentations of instability in the rugby player, which can be classified as: (1) frank dislocation requiring relocation, (2) subluxation and (3) subclinical instability.

1. Frank dislocation/dislocator

In this presentation, there is a definite report of shoulder dislocation, requiring relocation. Nowadays, most athletes present to the specialist after the first dislocation. It is imperative to know the exact mechanism of injury, as this will intimate the probable pathologies. The ease of relocation, method and sedation will also intimate the probable pathologies. The more difficult the relocation, the more significant the pathology. Lesions such as bony Bankart fractures, HAGL tears and rotator cuff tears should be expected.

2. Subluxation(s)

The player will have had single or recurrent episodes of true subluxations. They report that they felt their shoulder “slip out and then back in” on impact or tackle. It is important to ascertain the mechanism, for direction, and also the force of injury. The force of injury reflects the severity of pathology and also may reflect underlying hyperlaxity. Feelings of a temporary ‘dead arm’ at the time of injury, similar to neurogenic stingers, are commonly reported.

3. Subclinical instability

An injury may result in severe pain and ‘dead arm’, but no true dislocation or subluxation. The player may be able to continue the match, with severe post-match pain. They may be able to return to play, but sustain repeated episodes of pain and ‘dead arm’ with tackles and impact. Many are able to play through the season, but struggle with weight training, particularly pressing and overhead exercises. Painful clicking, clunking or popping from the joint are frequent.

### Clinical examination

If the patient presents within a few days of the initial injury, the examination is often limited by pain. Injecting the joint with local anaesthesia may assist the examination procedure and relieve pain. It is essential to assess the neuromuscular status, particularly the axillary nerve. ACJ assessment is important as this may be an associated injury or an exacerbation.

Later presentations are easier to examine, but often many of the standard instability tests are less reliable in large muscular athletes. In addition to the usual range of motion and rotator cuff strength tests, the following clinical tests are also useful:

1. Anterior instability
  - *Anterior apprehension test* [42] Often best performed with the patient seated or supine. The arm is brought gently and gradually into increasing range of abduction and external rotation. Rugby players often do not feel true apprehension, but may report ‘stretching’ and posterior pain (possibly from the bruised posterior humeral head impinging on the glenoid). Jobe’s relocation manoeuvre is often negative.
  - *Antero-inferior apprehension sulcus* The patient is asked to bend forward at the waist by about 30° and the arm dangling down. Downward traction on the arm can reproduce anxiety, discomfort or apprehension. This indicates antero-inferior instability and can be performed in varying positions to indicate the direction of antero-inferior instability.
2. Posterior instability
  - *Wrightington posterior instability test* (*WPIT*) [31] Posterior instability can be difficult to diagnose in large, muscular athletes. The usual posterior instability tests (Jerk and posterior apprehension tests) are only positive in patients with gross posterior instability. The patient’s arm is positioned in flexion and full adduction with internal rotation. The scapula is fixed. The patient is then asked to resist downward pressure at the wrist. Inability to maintain the flexion against resistance with posterior shoulder pain is a positive test for subtle posterior instability. This is different to a positive O’Brein’s test where there is anterior shoulder pain suggestive of a SLAP tear. The weakness is probably due to posterior translation of the humeral head and posterior cuff inhibition in full internal rotation and adduction of the shoulder.
  - *Kim’s test* [21] This is useful for overt posterior instability. With the patient seated, the arm is held by the examiner in a position of 90° abduction. The arm is brought into flexion and internal rotation with the examiner applying an axial load by pushing on the flexed elbow and with the other hand pushing the upper arm posteriorly. Posterior shoulder pain denotes a positive test.
3. Labral tears
  - *Modified dynamic labral shear test* [20] This is a useful, non-specific test for detecting possible labral tears in athletes. The arm is brought into abduction and loaded axially through the flexed elbow. Whilst maintaining axial load, the shoulder is circumducted. A positive painful click or clunk is suggestive of a labral tear.
4. Laxity
  - *Glenohumeral laxity* This should be assessed using the load-and-shift tests [11] in anterior, posterior and inferior (sulcus) directions, comparing to the opposite shoulder. The Gagey inferior laxity test [15] is also useful for any abnormal inferior capsular laxity.
  - *Generalised laxity* The Beighton score [4] is used to determine the degree of generalised ligamentous joint laxity. A score above 6/9 is indicative of generalised hypermobility, even in a large, muscular athlete.

### Investigations

**Fig. 2:**
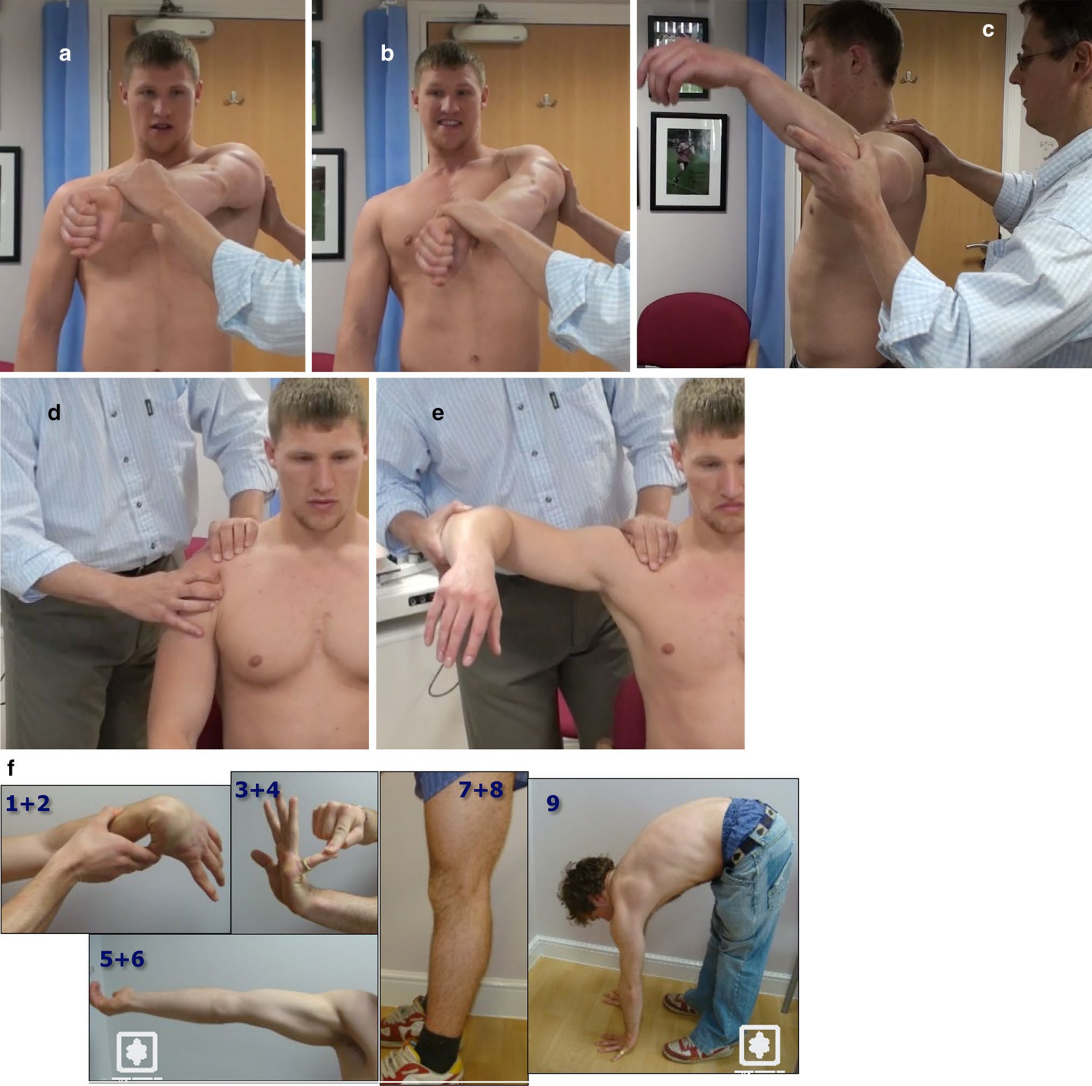
Useful clinical tests for rugby players: **a** and **b** WPIT (Wrightington posterior instability test) [31]: inability to maintain the arm in flexion and adduction against resistance with the scapula corrected. **c** Modified dynamic labral shear test [20] for labral tears. Axial load applied whilst circumducting the arm. **d** Load-and-shift tests [41] for glenohumeral joint laxity. **e** Gagey test for inferior capsular laxity [15]. **f** Components of the Beighton score [4] on a young rugby player

In the acute setting, radiographs are useful to diagnose any overt bony injuries; otherwise, an MRI scan within 24 h of the acute injury is useful to show most major soft tissue pathologies. Where there is blood in the glenohumeral joint, an arthrogram is not required. If unsure, an ultrasound scan may show a joint effusion, confirming blood in the joint (Fig. 2).

After 24 h from the acute injury, an MR arthrogram is generally the investigation of choice. It should show all major bony and soft tissue injuries. In addition to the standard sequences, additional fat-suppression sequences in the coronal plane and bone-enhancing T1 sequences in the axial and sagittal-oblique planes should be included. The fat-suppression sequences highlight any bone oedema, particularly around the ACJ and posterior humeral head. Bone-enhancing T1 sequences are helpful to diagnose small bony glenoid injuries that can often be missed and negate the need for a CT scan. ABER (Abduction external rotation) sequences are also useful to highlight labral and partial-thickness rotator cuff tears, as long as the patient is able to raise their arm overhead comfortably.

**Fig. 3:**
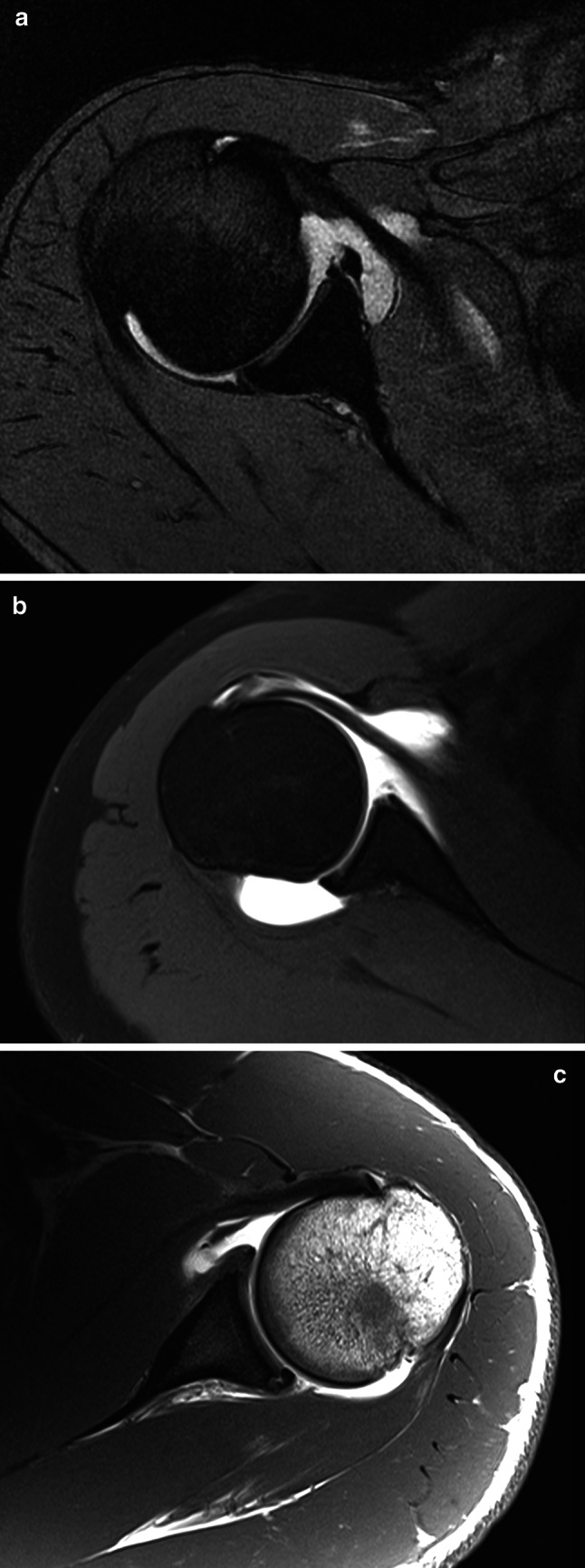
MR arthrogram images of labral injuries in rugby players, showing **a** a displaced anterior labral Bankart tear, **b** anterior bony glenoid lesion and **c** posterior labral tear

A CT arthrogram can be performed instead of an MR arthrogram. Traditionally, this is reported as being better for detecting bony lesions. CT scans are useful for any complex bony pathology (Fig. 3).

## Management

Management decisions in professional athletes are often stressful for all concerned. The athlete feels vulnerable and naturally concerned about their career. The club and national team are anxious about the athletes return to sport, and the team clinicians have a huge burden of responsibility. The surgeon’s role is to appreciate these issues in addition to managing the injury, based on their experience and knowledge of the published evidence. The clinical findings and imaging form a basis for the decision-making, but it is important to realise that these are a ‘snapshot in time’, and the patient’s functional ability to perform and pain are the key determinants on the degree of intervention needed.

The frequency, aetiology, direction, severity (FEDS) classification system highlight the factors to consider when deciding on surgery for shoulder instability [25]. Lebus et al. [25] showed that aetiology and increased frequency of instability are the strongest predictors for requiring surgical stabilisation. My algorithm for managing a rugby player, or any other athlete, is based on three factors:

1. Type of instability—frank dislocation(s), subluxation(s) or subclinical instability
2. Severity of structural, pathological injury—e.g. undisplayed labral tear versus large bony Bankart lesion
3. Time of season and career

1. Type of instability
  1.1. Frank dislocation/dislocator
    In a rugby player with a frank dislocation, significant injuries are often sustained due to the forces required to dislocate a large, muscular shoulder. It is also recognised that the recurrence rate following true dislocations in elite, contact athletes is high [1, 22, 32]. This is reduced significantly with surgical stabilisation [8, 24, 26, 29].
  1.2. Subluxation(s)
    The majority of athletes in this group are often able to return to play with little lost time from competition after a subluxation ‘instability event’ [12]. The structural injuries are generally less severe than a complete dislocation. Dickens et al. [12] showed that almost one-third of athletes complete the season without any instability episodes. Therefore, in-season surgery for this group is often not needed, depending on their pain and functional ability.
  1.3. Subclinical instability
    As with subluxations, these are ‘instability events’ that often do not prevent play. There is usually less severe structural injuries sustained. Although immediate surgery is not often required, progressive re-injury and progression of structural damage may require surgical repair when the athlete becomes functionally affected.
2. Structural injury
  The amount of glenohumeral joint damage is related to the risk of recurrence, with more significant lesions associated with higher risk of recurrence and a reduced ability to return to sport without surgical correction [30, 36]. These significant ‘major’ lesions are bony Bankart injuries, HAGL tears, large Hill–Sachs lesions and full-thickness rotator cuff tears. Any of these injuries would suggest a reduced ability to return to sport without surgical stabilisation and a high risk of recurrence [30, 37].
  ‘Minor lesions’ are often not associated with true dislocations and are less severe. These include isolated labral tears, partial-thickness rotator cuff tears and small Hill–Sachs lesions. However, hyperlaxity should be considered, as a hyperlax athlete may have a higher predisposition to re-injury with minor lesions [7, 39] (Table 1).

**Table 1:** Minor and major pathological lesions

Major lesions	Minor lesions
Bony Bankart	Labral tear
Full-thickness rotator cuff tear	Partial-thickness rotator cuff tear
Large Hill–Sachs lesion	Small Hill–Sachs lesion
HAGL tear
3. Timing
  The aim of treatment is to return the athlete back to sport as soon and as safely as possible, without jeopardising their long-term prognosis. There is contradictory evidence to suggest that surgery either increases or reduces the long-term risk of shoulder joint degeneration. Therefore, the primary aim of treatment is to treat the patient’s pain and functional limitations. It is often quicker to return an athlete to play without surgery for minor lesions without true dislocations [6, 12, 30, 37]. However, it is probably unsafe to do so for major lesions with true dislocations. Therefore, my approach is to offer in-season surgery to the latter group and try return to play in the former group, whilst taking into account the type on the severity of structural pathologies.

### Types of surgery

Surgery should aim to repair or reconstruct the pathological, structural lesions. Whether the approach is via open or arthroscopic surgery is less important. Almost all procedures can be performed via either route, with pros and cons depending on the surgeon, facilities and techniques.

#### Anterior instability surgery

Most capsulo-labral injuries without bony involvement and rotator cuff tears are repaired with suture anchors arthroscopically. Results are excellent in rugby players, without ‘major’ capsulo-labral lesions, such as HAGL and bony Bankart injuries.

Small, acute bony Bankart lesions can be successfully repaired if performed within 3 months of the original injury [28, 34, 38]. Chronic bony Bankart lesions have a high failure rate with direct repair, and a reconstructive procedure is recommended [34, 38].

HAGL tears are impossible to repair arthroscopically in the acute scenario, due to fluid extravasation. Arthroscopic repair of chronic HAGL tears is possible, but less likely to be successful in collision athletes [23]. Reconstructive surgery, in the form of the Latarjet procedure, is generally preferred.

#### Latarjet procedure

Chronic bony Bankart lesions progress to anterior bony glenoid deficiency with repeated instability. In rugby players, a bony reconstruction with coracoid transfer and sling effect of the modified Latarjet procedure is increasingly popular [5]. The degree of bony glenoid deficiency in isolation for Latarjet procedure is approximately 20 % glenoid bone loss. However, in a collision athlete, any degree of bone loss is significant, considering the forces involved and associated injuries. The instability severity index score (ISIS) [3] is a useful clinical tool to use for decision-making for a Latarjet procedure in athletes. It takes into account and highlights the patient’s age, hyperlaxity, glenoid bone involvement, Hill–Sachs lesion and level of sports participation (Table 2). It has been independently validated [33, 36], with a score of 4 and above indicative of a high failure rate of arthroscopic Bankart repairs.

**Table 2:** Instability severity index score (ISIS) [5]

Prognostic factor	Score
Age at surgery (years)	
<20	2
>20	0
Glenoid loss of contour on AP radiograph	
Loss of contour	2
No loss of contour	0
Hill–Sachs lesion on external rotation AP radiograph	
Visible	2
Not visible	0
Degree of sports participation	
Competitive	2
Recreational or none	0
Type of sport	
Contact or forced overhead	1
Other	0
Shoulder hyperlaxity	
Present	1
Not present	0
Total	10

#### Posterior instability surgery

Posterior labral involvement is more frequent in collision athletes than non-athletes [40]. It is more difficult to detect clinically as posterior instability generally presents with pain and clicking (subclinical) rather than true dislocation. Posterior labral tears are also more difficult to detect on imaging than Bankart tears, with a sensitivity under 60 %, but specificity of over 90 % [19]. Surgical repair can be more challenging than anterior repairs, but the principles are the same, with excellent results in rugby players without glenoid bone loss [2]. In cases of posterior glenoid bone loss with instability, posterior glenoid bone reconstruction with autograft, combined with capsulo-labral repair, may be required.

### Rehabilitation

The aim of rehabilitation after injury or surgery is to return the athlete to their previous level of sport as quickly and safely as possible. Adequate tissue healing to withstand the forces of the sport is required before returning safely to play. Our current basis for tissue healing times is inferred from experimental studies on laboratory animals and skin healing. Healing and recovery of an elite athlete may be a lot quicker than that, but currently we have no good way of measuring it. Traditionally, surgeons have used a time-based approach from empirical knowledge. As long as the surgical fixation is strong, the repair does not undergo undue strain within comfortable ranges of motion. The integrity of repairs should be tested on table determining a ‘safe-zone’ that therapists can use as a guide for post-operative safe, early motion. Only where there is a tendon repair, limit early active and resisted exercises. Elite athletes tend to have better conditioning, muscles and healing capacity than non-athletes. They also have a much higher level of therapy input, good supervision and motivation to be compliant with the rehabilitation process.

A goal-based, customised programme for return to sport after shoulder surgery is preferred [27]. This is shown in Table 3. The rehabilitation should be player-specific and tailored to suit the player’s age, position, requirements, surgeon and therapists. It is not ‘accelerated’ or ‘aggressive’ or ‘time-specific’. It is also surgeon-specific and dependent on the quality and type of fixation achieved. Therefore, the protocol is a ‘guide’ and not a prescription. Communication between the player, therapists, training staff and surgeon are essential, with progression to each phase when the patient is able to perform all of the exercises in the previous phase without any discomfort or apprehension. Each phase is introduced progressively.

**Table 3:** Rugby-specific rehabilitation programme

Phase 1	Level 1–2 exercises
Safe range of motion	Active assisted and progress to active motion in safe zone (as determined at surgery)
Safe joint loading	Isometrics, closed chain work, scapular exercises, proximal trunk activation
Fitness and conditioning	Able to bike immediately No upper limb weights Run in water (hydropool) Short runs on treadmill as comfortable
Sports-specific	Ball-to-hand passing in safe zone with rugby ball
Phase 2	Commence when completed phase 1 (usually 3 weeks post-op). Level 2–3 exercises
Range of motion	Progress to full active range of motion as comfortable, no stretching
Joint loading	Open chain exercises with good glenohumeral joint control through range, rotator cuff exercises through pain free range, graded resistance isometric/concentric Strength: push/pull movements e.g. bench/seated row
Sports-specific	Increase ball-to-hand passing, light perturbation training
Phase 3	When completed phase 2 (usually 6 weeks post-op). Level 3+ exercises
Range of motion	Eccentric posterior to the scapula plane Gently push lateral rotation if still stiff
Joint loading	Commence upper limb weights with conditioning coach Approx 50 % pre-op strength bench press/shoulder press Scapula plane abduction 50 % LSI Proprioception: single arm prone hold 50 % LSI; two point kneel on wobble cushion 75 % LSI Swimming freestyle if no risk of impingement
Sports-specific	Specific perturbation training exercises General skills passing, fending to specifics, throwing in, scrum half pass, catching the high ball etc.
Phase 4	Usually at 8 weeks post-op
Joint loading	Once 75 % pre-op strength bench/shoulder press/chin up/dumbell row = Commence power lifting/plyometrics
Sports-specific	Begin conditioning games and short training games Controlled Tackling/scrumming/lineout lifting (usually 10–16 weeks)
Phase 5	Return to play (usually 12–16 weeks) Graduated return to play Goals: Regained full pre-op strength in weights RC strength 90 % LSI. Objective assessment e.g. isokinetics Proprioception *R* = *L*

*LSI* limb symmetry index—percentage comparison of an activity to the opposite side; exercise levels are based on Funk et al. [14]

### Return to play

**Fig. 4:**
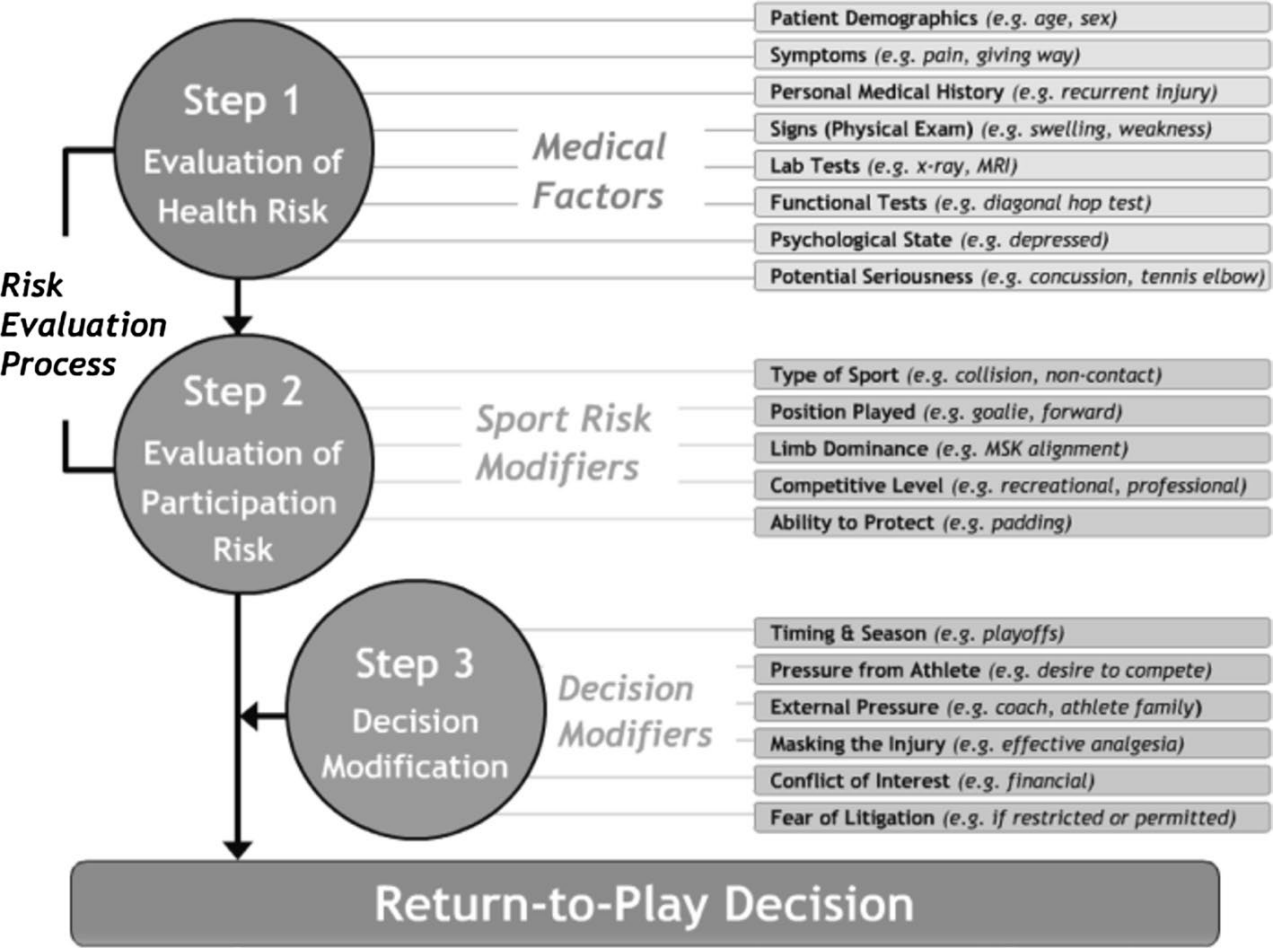
Return to play decision model [40]

The decision to return to full sport is based on the achievement of the sport-specific goals and dependant on a number of factors that influence the risk of recurrent injury. The decision model of Creighton et al. [9] is a useful guide to assisting and understanding and optimising this process. The three-step decision-based model comprises health status, participation risk and decision modification. This is demonstrated in Fig. 4.

Step 1, Health Risk: the health status of the athlete is assessed through the evaluation of medical factors related to how much healing has occurred. This is often based on clinical signs, imaging and the ability to achieve specific functional tests.

Step 2, Participation Risk: the clinician evaluates the participation risk associated with participation, which is informed by not only the current health status but also by the sport risk modifiers (e.g. ability to protect the injury with padding, athlete position). Different individuals are expected to have different thresholds for “acceptable level of risk”, and these thresholds will change based on context.

Step 3, Decision Modification: decision modifiers, such as timing and season, club and athlete pressures, are considered and the decision to return to play or not is made.

## Conclusion

There is a range of severity of degree of instability, direction and structural injuries in rugby. These are generally consistent and predictable based on an understanding of the injury mechanism, targeted examination and relevant imaging. The decision to operate is based on number of clinical and sports-specific factors. The decision on when to operate may be influenced by external factors. Therefore, the management of the rugby player with shoulder instability is often multidisciplinary and multifactorial. In this article, the current evidence and experience have been summarised to aide clinicians managing a collision athlete with shoulder instability, but each injured athlete should be managed as an individual.
